# Identification of a Novel Epigenetic Signature CHFR as a Potential Prognostic Gene Involved in Metastatic Clear Cell Renal Cell Carcinoma

**DOI:** 10.3389/fgene.2021.720979

**Published:** 2021-09-01

**Authors:** Xiangling Chen, Jiatian Lin, Qiaoling Chen, Ximian Liao, Tongyu Wang, Shi Li, Longyi Mao, Zesong Li

**Affiliations:** ^1^Guangdong Provincial Key Laboratory of Systems Biology and Synthetic Biology for Urogenital Tumors, Department of Urology, The First Affiliated Hospital of Shenzhen University, Shenzhen Second People’s Hospital (Shenzhen Institute of Translational Medicine), Shenzhen, China; ^2^Shenzhen Key Laboratory of Genitourinary Tumor, Department of Urology, The First Affiliated Hospital of Shenzhen University, Shenzhen Second People’s Hospital (Shenzhen Institute of Translational Medicine), Shenzhen, China; ^3^Shenzhen Institutes of Advanced Technology, Chinese Academy of Sciences, Shenzhen, China; ^4^Department of Minimally Invasive Intervention, Peking University Shenzhen Hospital, Shenzhen, China; ^5^NO.6 Middle School of Changsha, Changsha, China

**Keywords:** clear cell renal cell carcinoma, metastasis, CHFR, methylation, epigenetic

## Abstract

Metastasis is the main cause of clear cell renal cell carcinoma (ccRCC) treatment failure, and the key genes involved in ccRCC metastasis remain largely unknown. We analyzed the ccRCC datasets in The Cancer Genome Atlas database, comparing primary and metastatic ccRCC tumor records in search of tumor metastasis–associated genes, and then carried out overall survival, Cox regression, and receiver operating characteristic (ROC) analyses to obtain potential prognostic markers. Comprehensive bioinformatics analysis was performed to verify that the checkpoint with forkhead associated and ring finger domains (*CHFR*) gene is a reliable candidate oncogene, which is overexpressed in ccRCC metastatic tumor tissue, and that high expression levels of *CHFR* indicate a poor prognosis. A detailed analysis of the methylation of *CHFR* in ccRCC tumors showed that three sites within 200 bp of the transcription initiation site were significantly associated with prognosis and that hypomethylation was associated with increased *CHFR* gene expression levels. Knockdown of *CHFR* in ccRCC cells inhibited cell proliferation, colony formation, and migration ability. In summary, our findings suggest that the epigenetic signature on *CHFR* gene is a novel prognostic feature; furthermore, our findings offer theoretical support for the study of metastasis-related genes in ccRCC and provided new insights for the clinical treatment of the disease.

## Introduction

Renal cell carcinoma (RCC) is the most common (∼90%) and lethal type of kidney cancer, with clear cell RCC (ccRCC) being the most prevalent and aggressive subtype (∼75%) (Hsieh et al., 2017; Capitanio et al., 2019). Surgical excision for localized RCC, in the form of partial or radical nephrectomy, offers the chance of cure in these patients. However, approximately 30% of the patients show local recurrence or distant metastasis, along with a poor 5-year survival rate (Kunath et al., 2017; Wiechno et al., 2018). The most common sites of RCC metastasis are lungs and bones (Ho et al., 2017), and metastasis is the main reason of mortality associated with RCC. Thus, identification of the molecular characteristics underlying ccRCC tumor metastasis is urgently needed. Transcriptional profiling is an effective tool for discovering the molecular mechanisms underlying the metastasis or progression of ccRCC and predicting clinical outcomes. A comprehensive overview of the transcriptomic profiles of ccRCC was available from The Cancer Genome Atlas (TCGA) project. Using these data, we identified genes that support ccRCC metastases by comparing the gene expression profiles of metastatic tumors and primary tumor. Our study aimed to identify more genes associated with ccRCC tumor metastasis, thereby supporting the development of new gene targeted drugs for aggressive ccRCC.

We identified 4,933 differentially expressed genes (DEGs) between ccRCC tumor tissues and normal tissues as reported in TCGA and 86 metastatic phenotype-associated genes. To obtain the interactions between these 86 DEGs, we constructed a protein–protein interaction (PPI) network and obtained 22 seeds, of which 13 genes were associated with overall survival (OS). The checkpoint with forkhead-associated and ring finger domain (*CHFR*) gene stood out. It encodes the E3 ubiquitin ligase enzyme, an important checkpoint protein, which has been reported to inhibit tumorigenesis in a variety of tumors (Privette and Petty, 2008; Sanbhnani and Yeong, 2012). CHFR plays an important role in cell cycle regulation by delaying entry into metaphase in response to microtubular stress, by affecting substrates *via* both proteasome-dependent and independent process. CHFR could act as an E3 ubiquitin ligase that ubiquitinates and degrades the substrates. Yu et al. (2005) found that CHFR is a tumor suppressor that ensures chromosomal stability by controlling the expression levels of key mitotic proteins, such as Aurora A. Oh et al. (2009) reported that CHFR binds and downregulates HDAC1 by inducing its polyubiquitylation both *in vitro* and *in vivo* to suppress tumorigenesis. Other substrates of CHFR include, but are not limited to Kif22 (Maddika et al., 2009), PLK1 (Kang et al., 2002), poly(ADP-ribose) 1 PARP1 (Kashima et al., 2012), and TOPK (Shinde et al., 2013). CHFR can also target proteins not for degradation but to activate signal transduction. For example, CHFR binds to MAD2 to enable its activation and transport to the kinetochore; MAD2 is not able to inhibit anaphase progression without the help of CHFR, so CHFR abnormalities result in mitotic defects (Keller and Petty, 2011). Recent studies have shown that mitotic abnormalities are closely related to tumorigenesis (Funk et al., 2016). Thus, substantial evidence suggests that the mitotic checkpoint protein CHFR could serve as a biomarker for tumorigenesis, as well as a potential therapeutic target (Derks et al., 2014).

DNA methylation is found in the dinucleotides of nearly 80% of the CpG islands in the genome (Craig and Bickmore, 1994) and controls various cell functions, such as proliferation, differentiation, and apoptosis (Cao et al., 2020). In human cancers, the abnormal methylation of promoters could lead to the silencing or activation of target genes, affecting transcriptional pathways, and resulting in cancer development (Antequera and Bird, 1993). Genes can be regulated by methylation at a single site; for example, *CMTM3* is involved in the pathogenesis of testicular cancer, and is often silenced, at least partially, by methylation at a single specific CpG site in tumor tissue (Li et al., 2014). Fleischer et al. (2014) demonstrated the role of DNA methylation–based markers in clinical diagnosis and highlighted the importance of epigenetic changes in cancer. Some studies have shown that the downregulation of *CHFR* expression in some cancers is caused by hypermethylation of its promoter (Sanbhnani and Yeong, 2012; Derks et al., 2014). In ccRCC, *CHFR* hypermethylation is accompanied by elevated gene expression levels, but the cause is unknown. The classification of methylation states of *CHFR* may not be sufficiently detailed, and the specific sites associated with each category are unclear.

In our study, we found that *CHFR* can be used as a prognostic marker for malignant ccRCC, and the identification of three methylation sites near transcription initiation sites can predict patient prognosis by using comprehensive analysis; no previously identified markers can achieve this. Functional assays, including Cell Counting Kit 8 (CCK8), colony formation, and Transwell assays, indicate that CHFR is related to the malignant behavior of ccRCC cells. Taken together, our findings suggest that the epigenetic signature of *CHFR* is a novel prognostic gene involved in metastatic ccRCC.

## Results

### DEG Screening, Gene Ontology, Kyoto Encyclopedia of Genes and Genomes, and PPI Functions Analysis

**FIGURE 1:**
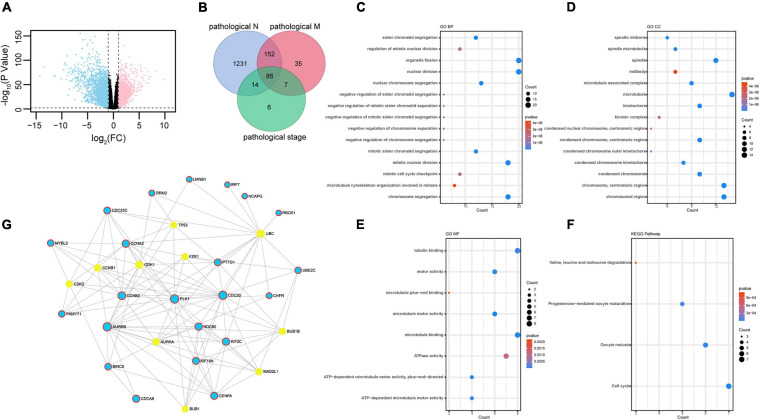
Identification of differently expressed genes: **(A)** 4,933 DEGs identified in ccRCC TCGA dataset plotted in the volcano plot, in which the logarithmic ratio of FC of the tumor/normal are plotted against negative logarithmic *P*-values; 2,571 genes were significantly downregulated (blue); 2,362 genes were upregulated (red) (| fold change| > 2, *p* < 0.01). **(B)** Venn diagram of metastasis-related genes in ccRCC in TCGA dataset. A total of 86 genes showed significant association with pathological M (*p* < 0.05), pathological N (*p* < 1E-5), and pathological stage (*p* < 1E-10). **(C–F)** GO terms representing BP, CC, and MF and KEGG pathway analysis. **(G)** PPI network of 86 DEGs. The 22 seeds genes are shown in blue. The STRING Interactome database was selected to construct PPI, and the confidence score cutoff was set as 900; degree filter threshold values > 5.

We selected DEGs in ccRCC normal and tumor tissues from TCGA database. Genes were considered upregulated or downregulated between normal and tumor tissues when their absolute fold change (tumor/normal) was greater than 2 (| FC| > 2) and their *p* value was less than 0.01 (*p* < 0.01). A total of 4,933 genes were identified as DEGs (Figure 1A and Supplementary Table 1). To obtain novel insight into the biology of metastatic ccRCC, the expression levels of 4,933 DEGs were compared further between the lymph node metastasis tissue (pathological_N1) and no lymph node metastasis tissue (pathological_N0), distant metastatic ccRCC tissues (pathological_M1) and primary tissues (pathological_M0), and different TNM stage tissues (pathological stage). Clinical characteristics are shown in Table 1. The Venn diagram showed that 86 genes were significantly associated with pathological N (*p* < 0.05), pathological M (*p* < 1E-5), and pathological stage (*p* < 1E-10) (Figure 1B and Supplementary Table 2). The 86 overlapped metastasis-associated genes were investigated using functional enrichment analysis, specifically Gene Ontology (GO) and Kyoto Encyclopedia of Genes and Genomes (KEGG). It is interesting that many cell cycle–related processes were enriched in biological process (BP), cell components (CC), molecular function (MF), and KEGG. This analysis indicated that in ccRCC metastatic process of ccRCC, cell cycle–related genes play important roles (Figures 1C–F). To obtain the interactions between the 86 DEGs, we constructed the PPI network using online Networkanalyst software. As shown in Figure 1G, the subnetwork included 32 nodes and 22 seeds. Detailed information concerning the seeds is provided in Supplementary Table 3.

**TABLE 1:** TNM clinical characters.

Characteristics		No. of patients
Pathological N	N0	240
	N1	17
Pathological M	M0	426
	M1	79
Pathological stage	I	269
	II	57
	III	125
	IV	84

### Survival Analysis of Hub Genes

**FIGURE 2:**
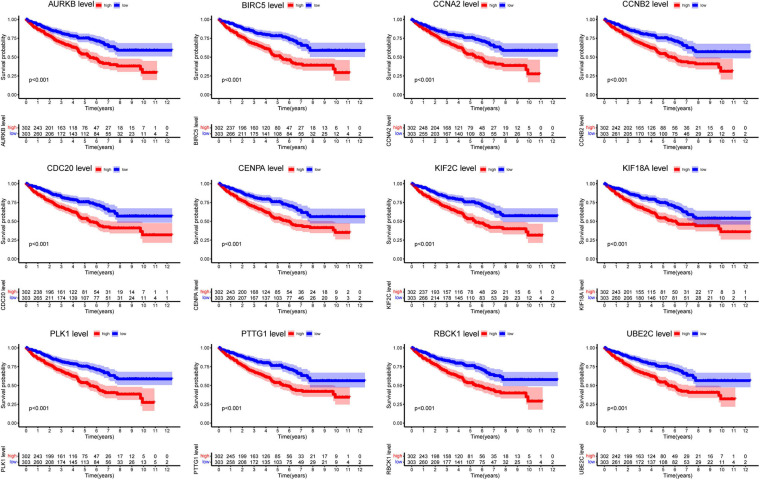
Overall survival analysis of all seed genes.

**FIGURE 3:**
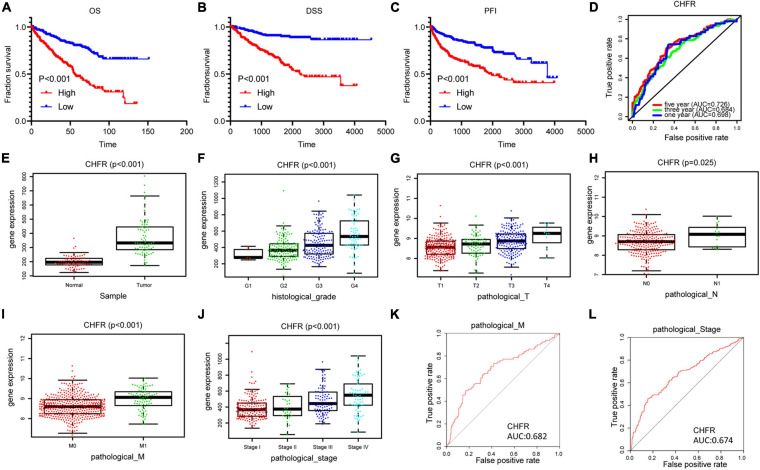
Identification of CHFR overexpression is associated with poor prognosis of ccRCC: **(A–C)** *CHFR* that was upregulated in ccRCC patients related to a shorter OS, PFI, and DSS survival based on TCGA dataset. **(D)** The time-dependent ROC for 1-, 3-, and 5-year OS predictions for the *CHFR* prognostic signature. **(E)** *CHFR* gene expression in ccRCC adjacent normal tissues and tumor tissues according TCGA dataset. **(F–J)** CHFR expression was analyzed in ccRCC patients regarding histological grade, pathological T, pathological N, pathological M, and pathological stage. **(K)** ROC curve analysis of *CHFR* as individual biomarkers to discriminate ccRCC patients with distant metastasis form patients without distant metastasis. **(L)** ROC curve analysis of *CHFR* as individual biomarkers to discriminate ccRCC patients in pathological stages III and IV from patients in pathological stages I and II.

Next, we analyzed all 22 seed genes associated with ccRCC from TCGA cohort. Thirteen genes were significantly associated with OS (*p* < 0.001) (Figures 2, 3A). Increased expression of all 13 genes correlated with higher risks, most notably in the case of *CHFR* (Figure 3A). The expression of *CHFR* in other cancers has also been investigated. Compared to the normal control, *CHFR* was also overexpressed in cancers, including bladder urothelial carcinoma, breast invasive carcinoma, cervical squamous cell carcinoma and endocervical adenocarcinoma, cholangiocarcinoma, glioblastoma multiforme, head and neck squamous carcinoma, kidney renal papillary cell carcinoma, liver hepatocellular carcinoma (LIHC), lung adenocarcinoma, lung squamous carcinoma, pheochromocytoma and paraganglioma, prostate adenocarcinoma (PRAD), and uterine corpus endometrial carcinoma (Supplementary Figure 1A). In addition, high *CHFR* expression was significantly associated with worse OS rates among PRAD, LIHC, and KIRC (ccRCC) patients (Figures 3A–C and Supplementary Figures 1B–D).

### CHFR Was Associated With Various Clinicopathological Variables

Next, we will focus on the clinical value of *CHFR* status in ccRCC. As previously mentioned, significantly worse OS rates, progression-free intervals (PFIs), and disease-specific survival (DSS) rates were observed in the ccRCC patients with high *CHFR* expression than those with low *CHFR* expression (Figures 3A–C). Univariate and multivariate Cox regression analyses showed that expression level could serve as an attractive predictor of prognosis in metastatic ccRCC patients (Table 2). Time-dependent receiver operating characteristic (ROC) analysis was used to determine the diagnostic value of *CHFR* expression, and the results showed that *CHFR* areas under the curves (AUCs) for the 1-, 3-, and 5-year OS prediction were 0.698, 0.684, and 0.726, respectively (Figure 3D). Our systematic analysis indicated that *CHFR* is a reliable prognostic gene in ccRCC. Next, we explored the role of *CHFR* in ccRCC metastasis. Based on TCGA datasets, *CHFR* gene expression levels were significantly upregulated in tumor tissue compared with those in adjacent control normal tissue (Figure 3E). In many other datasets, we obtained similar results; CHFR gene expression level was significantly upregulated in kidney tumor tissue compared with adjacent control normal tissue, as shown in Supplementary Figure 2. The TCGA dataset revealed that *CHFR* was highly expressed in the tissues of patients with higher tumor histological grades (Figure 3F), advanced primary tumor pathological stages (Figure 3G), lymph node metastasis (Figure 3H), distant metastasis (Figure 3I), and advanced pathological stages (Figure 3J). Moreover, ROC analysis was performed to evaluate the diagnostic accuracy of *CHFR* in differentiating the metastatic-related clinical characteristics of ccRCC patients. As expected, the results showed that *CHFR* was highly accurate in discriminating distant metastasis from no distant metastasis as well as advanced stages (III and IV) from early stages (I and II) (Figures 3K,L). These results indicate that *CHFR* can be used as a potential diagnostic parameter to distinguish high-risk from low-risk ccRCC patients.

**TABLE 2:** Univariate and multivariate Cox analyses.

Univariate analysis					Multivariate analysis			
Characteristics	HR	HR.95L	HR.95H	*p*	HR	HR.95L	HR.95H	*p*
Histological grade 1 + 2 vs. 3 + 4	2.634	1.876	3.700	<0.001				
Pathological T T1 + T2 vs. T3 + T4	3.179	2.107	4.797	<0.001				
Pathological N Yes vs. no	3.392	1.801	6.389	<0.001				
Pathological M Yes vs. no	4.351	3.190	5.935	<0.001	2.393	1.450	3.951	0.001
Pathological stage I + II vs. III + IV	3.550	2.307	5.461	<0.001	1.945	1.174	3.221	0.010
CHFR High vs. low	3.068	2.214	4.252	<0.001	3.402	2.093	5.530	<0.001

### Downregulation of CHFR Significantly Suppressed Proliferation and Migration of ccRCC Cells

**FIGURE 4:**
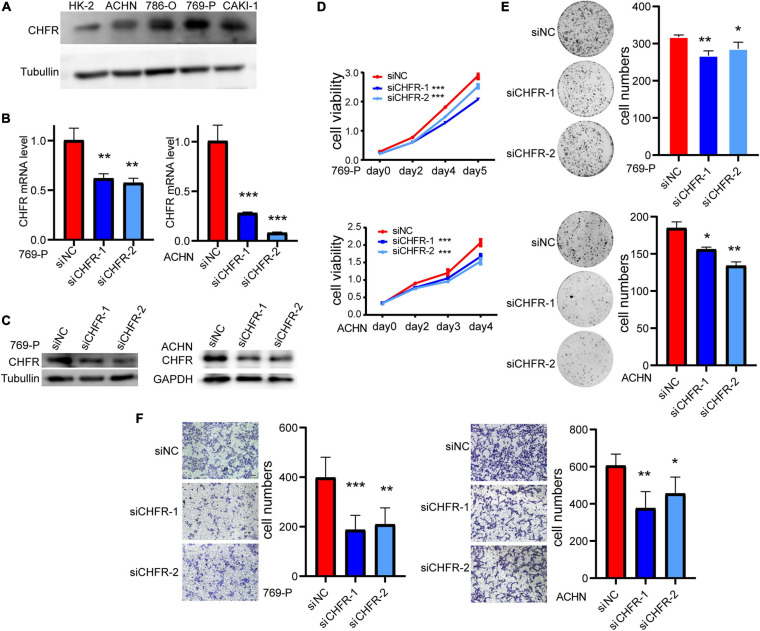
Depletion of CHFR suppresses ccRCC growth and metastasis: **(A)** Western blot: CHFR protein level was higher in ccRCC carcinoma cells than that in normal kidney cell line HK-2. **(B,C)** The knockdown efficiency of CHFR in 769-P and ACHN cells transfected with CHFR siRNA or control siRNA was verified by RT-PCR assay and Western blot assay. **(D)** CCK8 assay of ACHN and 769-P cells transfected with CHFR siRNA or control siRNA. The proliferation rates transfected with siCHFR were significantly lower than the control cells. **(E)** Colony formation assay. CHFR knockdown suppressed the colony formation. **(F)** Transwell assays were used to evaluate cell migration ability. Scale bar: 100 μm. All values are expressed as the mean ± SD (*p < 0.05, **p < 0.01, ***p < 0.001).

To clarify the expression of CHFR, we detected CHFR levels in ccRCC cells using Western blotting. The levels were upregulated in ACHN, 786-O, 769-P, and CAKI-1 ccRCC cell lines (Figure 4A). Real-time polymerase chain reaction (PCR) and Western blot assays established that *CHFR* mRNA and protein expression levels were significantly downregulated in 769-P and ACHN cells after treatment with targeted siRNAs (Figures 4B,C). The CCK8 assay indicated that cell proliferation was significantly lower for the cancer cells with *CHFR* knockdown than those with control siRNA transfection (Figure 4D). In addition, a significantly lower number of colonies were formed when *CHFR* was knocked down in 769-P and ACHN cells (Figure 4E). The results indicated that cellular proliferation capacity was suppressed when CHFR expression was reduced. The migratory ability of ccRCC cells in which CHFR deficiency occurred was also tested. Transwell analysis showed that knockdown of CHFR significantly inhibited the migration of 769-P and ACHN cells (Figure 4F). The above results demonstrated that CHFR had a critical effect on the proliferation and migration of ccRCC cells.

### Identifying Specific Prognostic Methylation Sites in CHFR

**FIGURE 5:**
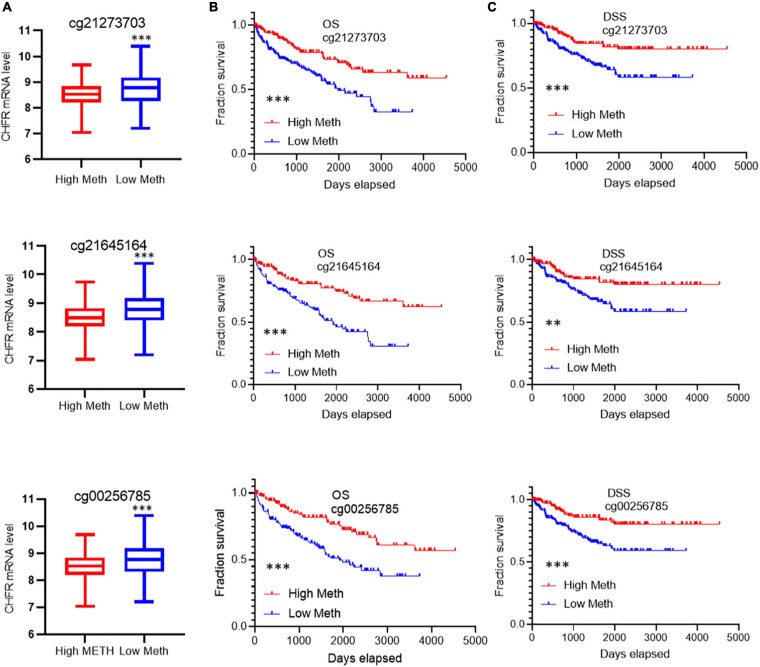
Correlation of the top 3 methylation sites with prognosis and *CHFR* expression: **(A)** Low levels of methylation at all three modification sites are associated with high gene expression **(B,C)** and associated with poor prognosis including overall survival and disease-specific survival *p < 0.05, **p < 0.01, ***p < 0.001.

*CHFR* expression levels have been reported to be regulated by methylation modifications. In ccRCC tumor tissues, although the overall methylation level of CHFR increased, the gene expression level was also significantly increased. Therefore, we analyzed the data related to methylation modification in TCGA database in detail and found that 73 sites on the CHFR gene were modified by methylation (Supplementary Table 4, methylation site and Cox analysis). Because DNA methylation in the promoter regions strongly influences gene expression, we selected CpGs in the promoter regions. Promoter regions were defined as 2 kb upstream to 0.5 kb downstream from the transcription start sites. Finally, we screened 23 methylation modification sites from these promoter regions (Supplementary Table 4, 23 TSS200-1500 sites). Next, in order to determine the methylation sites associated with survival outcomes, we selected prognosis-associated CpGs sites from the 23 methylation modification sites. We obtained 10 survival-associated methylation sites, of which seven predicted a poor prognosis when hypomethylated and three did so when hypermethylated (Supplementary Figure 3). Moreover, among these sites, only the top three were not based on optimal grouping, and these were within the range of 200 bp upstream of the transcription initiation region (Supplementary Figure 3). Therefore, we focused on the regulation of gene expression by these three prognostic sites. Patients were divided into hypermethylated and hypomethylated groups according to the three different prognostic sites’ methylation levels (calculated as reference ratios to medians). In all three sites, we found that the expression level of *CHFR* in the hypomethylated group was significantly increased (Figure 5A), and the hypomethylated group predicted a poor prognosis in terms of OS (Figure 5B) and DSS (Figure 5C). Therefore, the increase in *CHFR* gene expression levels in ccRCC may be mainly regulated by three methylation modification sites near the transcription initiation site, and our findings indicate their predictive and prognostic value as methylation-based biomarkers in the diagnosis and treatment of ccRCC.

## Discussion

Nearly 20% of ccRCC cases are at an advanced stage at the time of diagnosis (Mitchell et al., 2018). Even with surgical excision, 30% of localized ccRCC cases tend to show subsequent recurrence and metastases, and the 5-year survival rate of patients with distant metastases is approximately only 8–10% (Choueiri and Motzer, 2017; Miao et al., 2018). Therefore, there is an urgent need to further understand the molecular mechanisms that drive ccRCC metastasis to support the development of more effective therapeutic strategies. Rapid advances in genomics and transcriptomics have provided valuable opportunities to explore potential metastasis-related drivers.

In our study, in order to mine metastasis-related genes, we compared patient tissues with and without lymph node metastasis, tissues with and without distal metastasis, and tissues with high- and low-progression TNM stages, respectively, by using TCGA transcriptional profiling database. We suggest that genes that differ significantly in lymph node metastasis tissues, distal tumor metastasis tissues, and different pathological grades tissues are more likely to be markers of metastatic renal clear cell carcinoma, and thus, we further screened out the intersection genes of the three datasets. Through the above screening, a total of 86 genes were obtained, and the function and interaction analysis of 86 genes showed that, interestingly, many cell cycle–related genes were enriched. Many mitosis-related genes do play an important role in tumor development. For example, *UBE2C*, directly targeted by miR-548e-5p, increases cellular growth and invasive abilities of non–small cell lung cancer cells (Jin et al., 2019), *CDK1* interacts with *Sox2* and promotes tumor initiation in human melanoma (Ravindran Menon et al., 2018), and silencing CDCA8 suppresses hepatocellular carcinoma growth and stemness *via* restoration of the *ATF3* tumor suppressor (Jeon et al., 2021).

In particular, the checkpoint protein CHFR has attracted our attention. We found that CHFR was significantly overexpressed in ccRCC tissue samples with higher TNM grade and that CHFR overexpression predicted a poor prognosis and a higher risk of death. Several studies have suggested that CHFR expression is downregulated and inhibited by promoter hypermethylation in different types of cancer (Sanbhnani and Yeong, 2012; Derks et al., 2014). As a tumor suppressor gene, CHFR plays an important role in tumor progression and metastasis, as seen in gastric cancer (Hu et al., 2011), pancreatic cancer (Zhang et al., 2017), and esophageal adenocarcinoma (Soutto et al., 2010). We therefore evaluated the expression of *CHFR* in 33 different types of cancer from TCGA database and its relationship with prognosis. We found that *CHFR* was upregulated in many cancers, and high expression of *CHFR* in PRAD and LIHC also predicted poor prognosis. Our findings suggest a complex role for *CHFR* in different cancers. A detailed analysis of the methylation of *CHFR* at 73 sites in ccRCC tumors showed that three sites located within approximately 200 bp of the transcription initiation site were significantly associated with prognosis and that hypomethylation was associated with increased gene expression levels. We performed a series of functional experiments on ccRCC cells. The results showed that knockdown of *CHFR* inhibited the proliferation, invasion, and metastasis of ccRCC cells. Taken together, our findings suggest that the epigenetic signature of the *CHFR* gene is a novel prognostic feature involved in metastatic ccRCC.

Our study had a few limitations. First, we used public databases, and these findings need to be validated in prospective clinical trials. Second, although we have demonstrated the role of *CHFR* in ccRCC cells *in vitro*, its effect on ccRCC development needs to be further studied *in vivo*. Concurrently, it is necessary to explore the molecular mechanisms of *CHFR* in ccRCC. In conclusion, this study has increased our understanding of the metastatic mechanism in ccRCC, suggesting that *CHFR* expression can be used as a biomarker for the prognosis of ccRCC, although further study of the related molecular pathways of *CHFR* in ccRCC is needed.

## Materials and Methods

### Cell Culture

ccRCC cell lines were purchased from the Cell Bank of the Chinese Academy of Sciences (Shanghai, China); 769-P and 786-O cells were cultured in Roswell Park Memorial Institute 1640 (RPMI 1640); ACHN and HK-2 were cultured in MEM; and CAKI-1 was cultured in McCoy 5A. All cells were cultured with 10% fetal bovine serum (FBS) and 1% penicillin/streptomycin in an incubator at 37°C with 5% CO_2_.

### siRNAs and Antibodies

All siRNA sequences were synthesized by GenePharma (Shanghai, China). Two siRNAs of CHFR were: 5′-AGCCTT TCTGCCACCTGTATT-3′ (siCHFR_1), 5′-CCACAGCCATC AACATCGATT-3′ (siCHFR_2). RNAs were transfected at 60 nM with Lipofectamine 3000 (Thermo, L3000015). The CHFR, tubulin, and GAPDH antibodies were purchased from Proteintech (Wuhan, China).

### CCK8 Cell Proliferation Assay

Cells were seeded into 96-well plates (2,000 cells/well). CCK8 (Dojindo, Japan) was used according to the manufacturer’s instructions. After incubating cells for 2 h, we detected the optical density at 450 nm for each well by using a microplate reader.

### Colony Formation Assay

Cells were plated into six-well plates (2,000 cells/well). Cells were cultured for 14 days and then washed three times with phosphate-buffered saline (PBS), subsequently fixed with methanol, and then stained with 0.5% crystal violet solution for 20 min. The numbers of colonies of each well were manually counted.

### Transwell Migration Assay

Using Transwell chamber (8-μm pore size, Corning, United States) to perform cell migration assay. Cells were placed on the upper layer of cell-permeable membrane, in the lower chamber with 10% FBS media, incubated for 20 h, and then fixed with 4% paraformaldehyde, washed with PBS, and stained with 0.5% crystal violet; the upper chamber cells were removed. Cells that had passed through the pore and adhered to the lower membrane surface were counted.

### RNA Isolation and Quantitative Real-Time PCR

RNA was isolated from cells using Trizol reagent (Sigma, United States) (Rio et al., 2010). RNA was reverse transcribed into cDNA using the mRNA RT Reagent Kit (TaKaRa). Reverse transcriptase (RT)–PCR using FastStart Universal SYBR^®^ Green Master (ROX) (Roche, Germany) was carried out on an Applied Biosystems 7500 real-time PCR system. The gene ACTIN served as an endogenous control for normalization. The CHFR forward primer was as follows: GATGGTCACTCTGTCACCTGC, reverse primer: TTGTGGCTTCCCAGCATTGG; the ACTIN forward primer: CATCCGCAAAGACCTGTACG, reverse primer: CCTGCTTGCTGATCCACATC.

### Database

The gene expression profiles and clinical data of patients with ccRCC were obtained from UCSC Xena^1^ including 533 ccRCC cases in TCGA. The ccRCC cancer DNA methylation data were downloaded from the MethSurv data portal^2^ (named KIRC_meth.RData, 2017), The methylation level of each probe was represented by the *b*-value, which ranges from 0 to 1, corresponding to unmethylated and fully methylated, respectively. The TIMER online tool was used to analyze the expression of the CHFR gene in different tumors.^3^ The Networkanalyst software was used to analyze PPI network.

### Identification of DEGs

We identified the DEGs in ccRCC from TCGA according to the following cutoff value: *p* < 0.01 and | log2 fold change (FC)| > 1.

### GO and KEGG Pathway Enrichment

We used the overlapped DEGs and DEPs both in TCGA and CPTAC for gene enrichment and functional annotation analyses by “ClusterProfiler” packages in R 3.6.1.

### Survival Analysis

The survival R package was used to analyze the relationship between 22-hub-gene expression levels and the OS of patients from the TCGA dataset. SPSS was used to analyze the relationship between CHFR gene expression levels and PFI/DSS. We tested this relationship by Kaplan–Meier method with a log-rank test, where *p* < 0.05 was regarded as statistically significant.

### Establishment of Regression Model and Construction of OS Risk Prognostic Models

Univariate and multivariate Cox models were used to investigate the correlation between *CHFR* gene expression level and certain clinical characteristics, namely, histological grade, pathological T, pathological M, pathological stage, and OS, in ccRCC patients. Time-dependent ROC analysis for OS was used to evaluate the accuracy of the prognostic model. The correlations between the aforementioned clinical characteristics were also analyzed.

### Statistical Analysis

Statistical analyses were performed using SPSS Statistics software (version 23.0; IBM SPSS, Chicago, IL, United States), GraphPad Prism 8.0 (GraphPad Software, Inc., United States), and R 3.6.1. Functional and KEGG enrichment analyses were performed using the “ClusterProfiler” package. The time-dependent ROC analysis was performed using the “survivalROC” package. Kaplan–Meier analysis was performed to estimate the correlation between CHFR expression and OS, DSS, and PFI using the log-rank test. The prognostic significance of *CHFR* in ccRCC was analyzed using univariate and multivariate Cox proportional hazard regressions. To further evaluate the diagnostic value of *CHFR* mRNA expression, we generated ROC curves and calculated the AUC. All *in vitro* experiments were performed in triplicate or quintuplicate, and all data are represented as mean ± SD. Statistical significance was indicated by *p* < 0.05, and significance levels are shown as ^∗^*p* < 0.05, ^∗∗^*p* < 0.01, and ^∗∗∗^*p* < 0.001.

## Data Availability Statement

The original contributions presented in the study are included in the article/Supplementary Material, further inquiries can be directed to the corresponding author/s.

## Author Contributions

XC, JL, and QC performed the experiments. XC sorted out the article ideas and wrote the manuscript. JL and QC modified the article. SL and LM participated in the data analysis. XL and TW contributed to the wound-healing assays and invasion assays. ZL conceived the project and supervised the experiments. All authors contributed to the article and approved the submitted version.

## Conflict of Interest

The authors declare that the research was conducted in the absence of any commercial or financial relationships that could be construed as a potential conflict of interest.

## Publisher’s Note

All claims expressed in this article are solely those of the authors and do not necessarily represent those of their affiliated organizations, or those of the publisher, the editors and the reviewers. Any product that may be evaluated in this article, or claim that may be made by its manufacturer, is not guaranteed or endorsed by the publisher.

## Footnotes

1
https://xenabrowser.net/datapages/

2
https://biit.cs.ut.ee/methsurv/

3
http://timer.comp-genomics.org

## Supplementary Material

Supplementary Figure 1 Pan-cancer mRNA expression of CHFR. **(A)** Boxplots show *CHFR* expression in tumor (red) and normal (blue) tissue samples in different types of cancer. **p* < 0.05, ***p* < 0.01, ****p* < 0.001. **(B–D)** PRAD, LIHC, and KIRC patients with high *CHFR* expression had a worse OS rate than those with low *CHFR* expression.

Supplementary Figure 2 Identification of CHFR overexpression in kidney tumor tissues vs. normal tissues from different Oncomine datasets.

Supplementary Figure 3 Cox regression analysis of 23 methylation sites.
