# Supplementary figures and images for: Identification of a Novel Epigenetic Signature CHFR as a Potential Prognostic Gene Involved in Metastatic Clear Cell Renal Cell Carcinoma
Source: Front Genet. 2021 Sep 1;12:720979. doi: 10.3389/fgene.2021.720979 (PMC8440929; doi:10.3389/fgene.2021.720979)

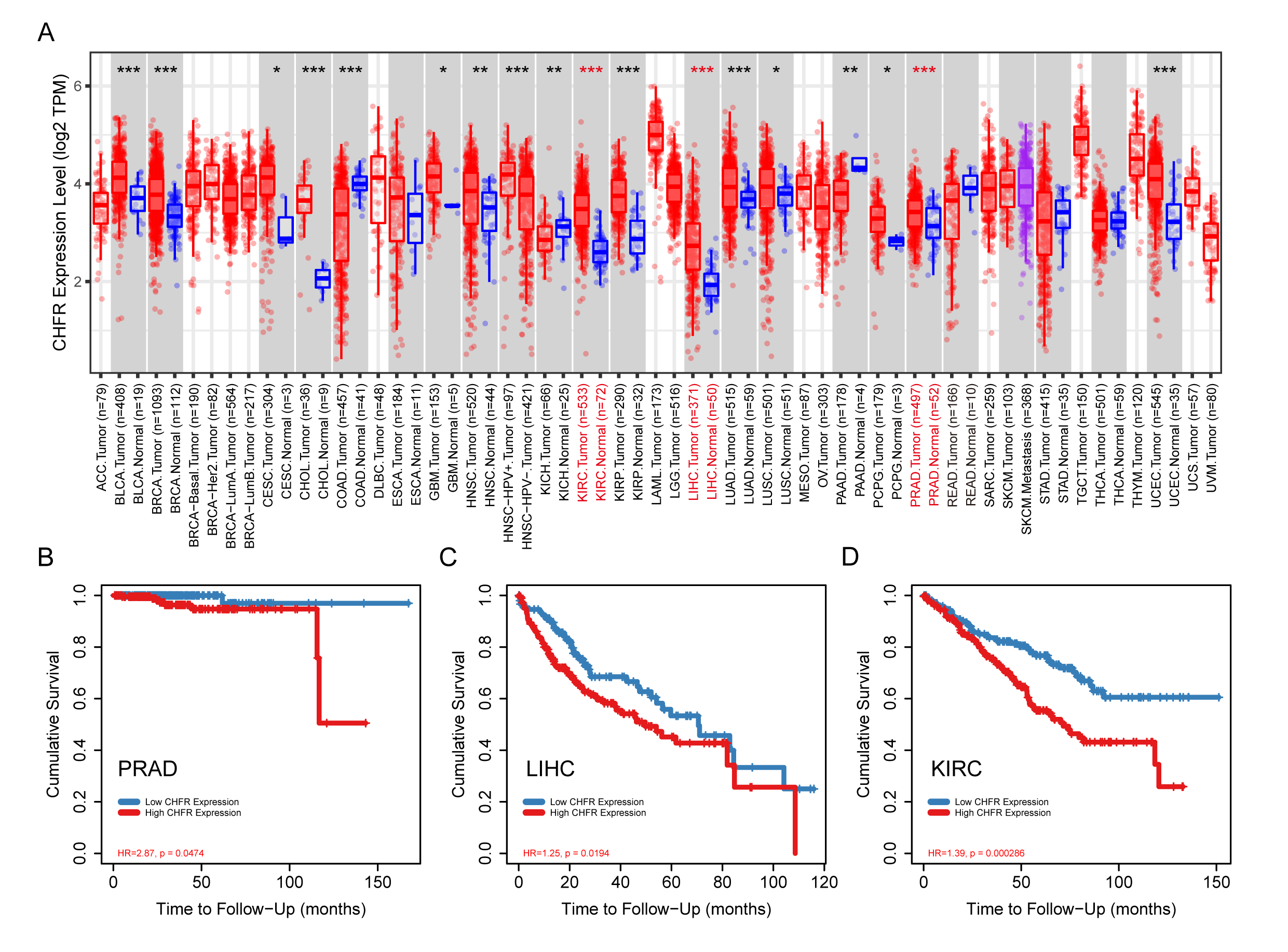

Supplement: Supplementary Figure 1 — Pan-cancer mRNA expression of CHFR. (A) Boxplots show CHFR expression in tumor (red) and normal (blue) tissue samples in different types of cancer. *p < 0.05, **p < 0.01, ***p < 0.001. (B–D) PRAD, LIHC, and KIRC patients with high CHFR expression had a worse OS rate than those with low CHFR expression. [file Image_1.TIF]

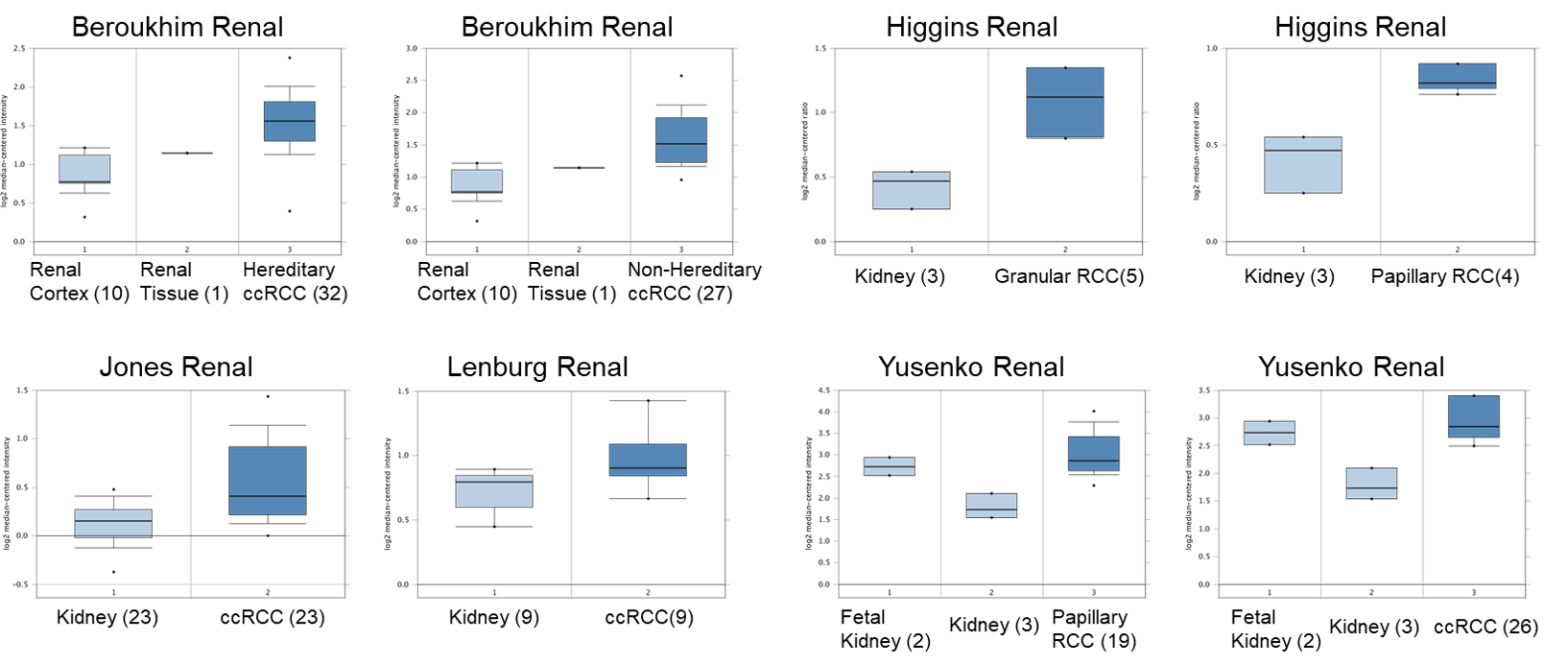

Supplement: Supplementary Figure 2 — Identification of CHFR overexpression in kidney tumor tissues vs. normal tissues from different Oncomine datasets. [file Image_2.TIF]

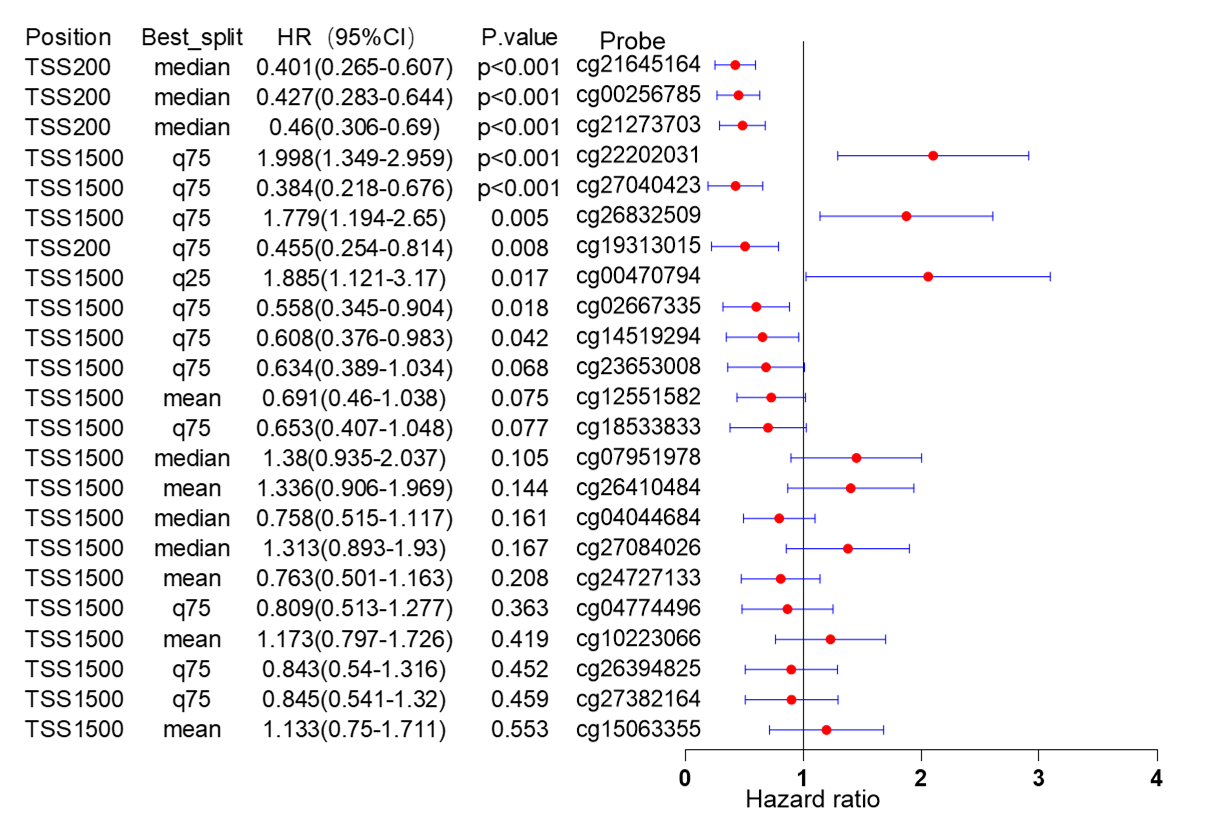

Supplement: Supplementary Figure 3 — Cox regression analysis of 23 methylation sites. [file Image_3.TIF]
